# The Stability Improvement of *α*-Amylase Enzyme from *Aspergillus fumigatus* by Immobilization on a Bentonite Matrix

**DOI:** 10.1155/2022/3797629

**Published:** 2022-01-10

**Authors:** Yandri Yandri, Ezra Rheinsky Tiarsa, Tati Suhartati, Heri Satria, Bambang Irawan, Sutopo Hadi

**Affiliations:** ^1^Department of Chemistry, Faculty of Mathematics and Natural Sciences, University of Lampung, Jl. Prof. Dr. Sumantri Brojonegoro No. 1, Bandar Lampung 35145, Indonesia; ^2^Department of Biology, Faculty of Mathematics and Natural Sciences, University of Lampung, Jl. Prof. Dr. Sumantri Brojonegoro No. 1, Bandar Lampung 35145, Indonesia

## Abstract

The stability of the *α*-amylase enzyme has been improved from *Aspergillus fumigatus* using the immobilization method on a bentonite matrix. Therefore, this study aims to obtain the higher stability of *α*-amylase enzyme from *A. fumigatus*; hence, it is used repeatedly to reduce industrial costs. The procedures involved enzyme production, isolation, partial purification, immobilization, and characterization. Furthermore, the soluble enzyme was immobilized using 0.1 M phosphate buffer of pH 7.5 on a bentonite matrix, after which it was characterized with the following parameters such as optimum temperature, Michaelis constant (*K*_*M*_), maximum velocity (*V*_max_), thermal inactivation rate constant (*k*_i_), half-life (*t*_1/2_), and the change of energy due to denaturation (Δ*G*_*i*_). The results showed that the soluble enzyme has an optimum temperature of 55°C, *K*_*M*_ of 3.04 mg mL^−1^ substrate, *V*_max_ of 10.90 *μ*mole mL^−1^ min^−1^, *k*_i_ of 0.0171 min^−1^, t_1/2_ of 40.53 min, and Δ*G*_*i*_ of 104.47 kJ mole^−1^, while the immobilized enzyme has an optimum temperature of 70°C, *K*_*M*_ of 8.31 mg mL^−1^ substrate, *V*_max_ of 1.44 *μ*mole mL^−1^ min^−1^, *k*_i_ of 0.0060 min^−1^, *t*_1/2_ of 115.50 min, and Δ*G*_*i*_ of 107.37 kJ mole^−1^. Considering the results, the immobilized enzyme retained 42% of its residual activity after six reuse cycles. Additionally, the stability improvement of the *α*-amylase enzyme by immobilization on a bentonite matrix, based on the increase in half-life, was three times greater than the soluble enzyme.

## 1. Introduction

Hitherto, microbial *α*-amylase is used commercially as biocatalysts in various industries, such as detergent, syrup, bread and cake, dairy products, starch processing, animal feed, textile and leather, pulp and paper, candy, sugar, bioethanol, pharmaceuticals, and waste treatment [1, 2]. Furthermore, it has a 25% demand in the global market [3]. The *α*-amylase enzyme (*α*-1,4-glucan-4-glucanohydrolase) breaks down the starch molecule by acting on the *α*-1,4-glucosidic bonds, thereby producing maltose, dextrin, or D-glucose [2]. However, despite its wide usage on the industrial scale, mostly enzymes are easily soluble in water when applied in batch processes, and this weakens the hydrogen bonds which contribute to stability [4]. The decrease in the stability of enzymes causes denaturation due to the loss of equilibrium in the noncovalent bonds [5], resulting in the once use of nonimmobilized enzymes. However, the price of commercial enzymes in the global market is quite expensive [6].

Immobilization is one of the simple ways to stabilize the enzyme structure, and it is the process of binding or retaining molecules to material or matrix which is insoluble in water [7]. Furthermore, one of the advantages of immobilized enzymes is that they are used repeatedly. In this study, the soluble *α*-amylase was immobilized on bentonite as an inorganic material and the smectite clay mineral with a structure consisting of two tetrahedral and one octahedral layer. The silicate of each tetrahedral layer interacts with the hydroxyl groups in the octahedral layer; hence, the tetrahedral-octahedral-tetrahedral (TOT) layers are formed. Furthermore, there are cations between the octahedral and tetrahedral layers that are involved in enzyme immobilization [8]. The characteristics of bentonite include insoluble in water, has large particle surface area, chemically inert, and thermally stable, has a well-defined layered structure, has the ion-exchange ability, abundant raw material, inexpensive, and eco-friendly [9]. Based on the previous study [10], the immobilized *α*-amylase from *Bacillus subtilis* ITBCCB148 on the bentonite from Sigma Aldrich™ has two-fold higher thermal stability than nonimmobilized enzyme, with residual activity of 43% after five cycles of reuse. In the present study, *Aspergillus fumigatus* was chosen as the host enzyme because this fungus produces the *α*-amylase without any special nutritional needs [11].

## 2. Materials and Methods

The local fungal isolate of *A. fumigatus* was obtained from the Laboratory of Microbiology, Department of Biology, Lampung University. Also, bentonite was purchased from Sigma Aldrich™. All chemicals and reagents used in this study were of analytical grade.

### 2.1. Study Procedure

The study procedures were based on a previous study, comprising production, isolation, partial purification, immobilization, and characterization of the soluble and immobilized enzymes [12]. Furthermore, the crude enzyme was soluble by fractionation using ammonium sulphate and dialysis as described in [13], while the immobilization method was based on [10]. The crude enzyme was partly refined by ammonium sulphate in an ice bath. The precipitated protein was collected by centrifugation at 5000 rpm for 15 min at 4°C and dissolved in a minimum volume of phosphate buffer (0.025 M; pH 6.5). Then, the enzyme suspension was dialyzed at 4°C against phosphate buffer (0.01 M; pH 6.5) for 24 h at 4°C [13].

### 2.2. Analysis of *α*-Amylase Activity and Determination of Protein Content

The *α*-amylase enzyme activity in the partial purification steps was analyzed by the Fuwa method using iodine reagent [14]. Meanwhile, the activity has been analyzed in characterization steps by Mandel's method using dinitrosalicylic acid reagent [15], whereby the protein content was determined based on the Lowry method [16].

### 2.3. Determination of Initial Buffer pH

The soluble enzyme was immobilized on a bentonite matrix using 0.1 M phosphate buffer with variations in pH which includes 5.0, 5.5, 6.0, 6.5, 7.0, 7.5, and 8.0. Furthermore, the bentonite matrices (0.20 g) were washed 2-3 times using each buffer by centrifugation at 5000 rpm for about 15 min until the pH was reached. 0.5 ml of the soluble enzyme and 0.5 mL of each buffer were then added to the matrices. Afterward, these samples were incubated at 4°C for 30 min and centrifuged for about 15 min. From these supernatants, 0.25 mL each was collected separately as “binding” and control samples. After which, the immobilized enzymes at pH 5.0–6.5 were then eluted from their matrix using a 1.0 mL mixture of 0.1 M phosphate buffer pH 8.0 and 1 M NaCl (1 : 1), while the immobilized enzymes at pH 7.0-8.0 were eluted from their matrix using 1.0 mL mixture of phosphate buffer 0.1 M pH 5.5 and 1 M NaCl (1 : 1). All samples were then centrifuged for up to 15 min, and from the supernatants, 0.25 mL was as the “elution” sample. The enzymatic activity was analyzed by the Fuwa method, whereby the pH that has both lower and higher activity in the binding and elution process was selected as the initial buffer pH in the immobilization procedure.

### 2.4. Immobilization of *α*-Amylase on a Bentonite Matrix

The bentonite (0.20 g) was washed 2-3 times using the initial buffer by centrifugation for up to 15 min until the pH was reached. 0.5 ml of the soluble enzyme and 0.5 mL of initial buffer were then added to the matrix. Afterward, it was incubated at 4°C for 30 min and centrifuged for about 15 min. From the supernatant, 0.25 mL was collected as a control. Subsequently, 0.75 ml of the starch substrate was added to the immobilized enzyme, which was incubated at its optimum temperature for 30 min, and centrifuged for about 15 min. The supernatant was assayed for *α*-amylase enzyme activity by Mandel's method.

### 2.5. Determination of Optimum Temperature

This was performed at different incubation temperatures in Mandel's assay which includes 50, 55, 60, 65, 70, and 75°C for 30 min. Therefore, the incubation temperature, which generates the highest enzyme activity, was used to determine the optimum temperature.

### 2.6. Steady-State Kinetics

Kinetic parameters, the Michaelis constant (*K*_*M*_) and the maximum velocity (*V*_max_), were calculated using the Lineweaver–Burk plot from experimental data on the effect of starch substrate concentration against the enzyme activity in the following concentration ranges: 0.2, 0.4, 0.6, 0.8, and 1.0%. The enzyme activity is proportional to the enzyme-catalyzed reaction rate [17]. Meanwhile, both were analyzed by Mandel's method at each optimum temperature, and the *K*_*M*_ values of soluble and immobilized enzymes were used as each optimum substrate concentration for subsequent procedures.

### 2.7. Determination of Thermal Stability

Thermal stability of the enzyme was determined from the residual activity after being inactivated at 60°C for the following variation of time (*t*_*i*_): 0, 10, 20, 30, 40, 50, 60, 70, and 80 min [18–20]. Furthermore, the enzyme activity was analyzed using Mandel's method. The data were also used to determine the values of *k*_*i*_, *t*_1/2_, and Δ*G*_*i*_.

### 2.8. Determination of *t*_1/2_, *k*_*i*_, and Δ*G*_*i*_

The thermal inactivation rate constant (*k*_*i*_) and half-life (*t*_1/2_) were calculated using the following first-order enzyme inactivation rate equation.

(1) $$\begin{array}{c} \ln\frac{E_{i}}{E_{o}}=-k_{i}\times t_{i}, \end{array}$$

where *k*_*i*_ is the thermal inactivation rate constant, *E*_*o*_ is the residual activity at *t*_*o*,_*E*_*i*_ is the residual activity at *t*_*i*_, and *t*_*i*_ is the inactivation time. The slope of a graph ln(*E*_*i*_/*E*_*o*_) against *t*_*i*_ was determined as the *k*_i_ value [21, 22].

The free energy due to denaturation (*G*_*i*_) is the energy required for the denaturation of the enzyme from the initial state. The Δ*G*_*i*_ value was calculated from the following derivation of thermodynamic equation.

(2) $$\begin{array}{c} \Delta G_{i}=-\;RT\;\ln\frac{k_{i}\cdot h}{k_{B}\cdot T}, \end{array}$$

where Δ*G*_*i*_ is the change of energy due to denaturation, *R* is the ideal gas constant, *T* is the thermal inactivation temperature (K), *k*_*i*_ is the thermal inactivation rate constant, *h* is the Planck constant, and *k*_*B*_ is the Boltzmann constant [21].

### 2.9. Reusability Study

The immobilized enzyme that reacted with the substrate was washed by centrifugation using the initial buffer, and from the supernatant, 0.25 mL was collected as a control sample. Furthermore, the immobilized enzyme was reacted with a new substrate (0.75 mL) [10, 23] and the activity was determined by Mandel's method. This procedure was performed repeatedly six times.

### 2.10. Statistical Analysis

All measurements were done in duplicate (*n* = 2), and data were reported as mean ± standard deviation (SD). Analysis of variance (ANOVA) accompanied by the student *t*-test (paired two samples for means) was conducted to identify the significant differences between two replicate samples. The level of significance was set at *p* < 0.05. The null hypothesis had been rejected, and there is no difference between the two replicate measurements.

## 3. Results and Discussion

### 3.1. Purification of *α*-Amylase

**Figure 1:**
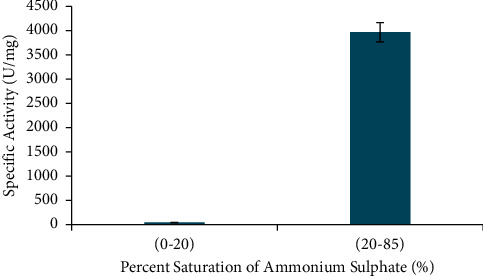
Ammonium sulphate fractionation scheme. The data are presented as mean ± SD; *n* = 2; a significant difference = *p* < 0.05. The error bars represent standard deviations from two replicates measurements.

The crude enzyme from *A. fumigatus* was partially purified by ammonium sulphate precipitation and followed by dialysis. The relationship between percent saturation of ammonium sulphate against the enzyme specific activity is shown in Figure 1. Based on Figure 1, the *α*-amylase yield was partly refined by 20–85% ammonium sulphate, and its specific activity was 3970.08 U/mg.

The result of partial purification steps of the *α*-amylase enzyme is given in Table 1. The outcomes in Table 1 indicated that the *α*-amylase yield of 345.57 and 179.68 U/ml was obtained from 20% to 85% ammonium sulphate and dialysis, respectively, in comparison to crude amylase of 100.37 U/ml. In addition, we observed increasing the specific activity of the enzyme that indicated an increase of its purity from each purification step. The purity of the enzyme increased ten-fold after dialysis. It was supported by the decrease in total protein and the yield (%) of the enzyme which indicated that the enzyme was free from impurities.

### 3.2. Determination of Initial Buffer pH

**Figure 2:**
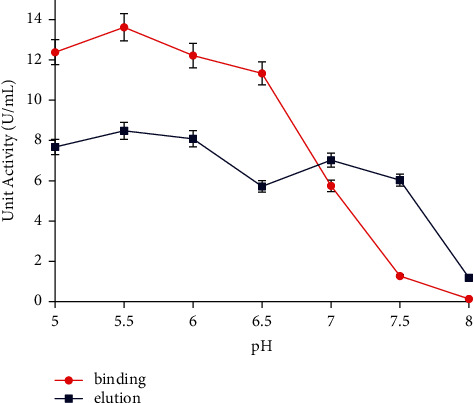
Binding buffer pH for enzyme immobilization. The data are presented as mean ± SD; *n* = 2; a significant difference = *p* < 0.05. The error bars represent standard deviations from two replicates measurements.

The *α*-amylase enzyme molecules were successfully bound to the bentonite matrix via physical adsorption at the alkaline pH range of 7.0–8.0. Furthermore, a pH 7.5 showed both the low and high enzyme activities in the binding and elution process, as shown in Figure 2. Hence, pH 7.5 was selected for the initial buffer.

### 3.3. Determination of Optimum Temperature

**Figure 3:**
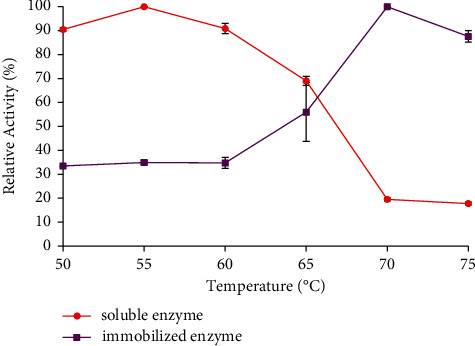
Optimum temperature of the soluble and immobilized enzymes. The data are presented as mean ± SD; *n* = 2; a significant difference = *p* < 0.05. The error bars represent standard deviations from two replicates measurements.

The temperature profiles of soluble and immobilized enzymes are shown in Figure 3. As shown in Figure 3, the optimum temperature of soluble and immobilized enzymes was 55°C and 70°C, respectively. However, this changes position due to space obstruction by the bentonite matrix against enzyme molecules which prevents denaturation. Thus, the immobilized enzyme is more stable than the soluble.

### 3.4. Steady-State Kinetics

**Figure 4:**
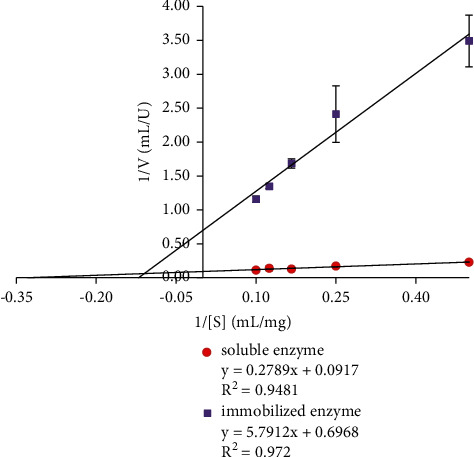
Lineweaver–Burk plot analysis of the enzymatic kinetics of soluble and immobilized enzyme. The values were presented as mean ± SD; *n* = 2; a significant difference = *p* < 0.05. The error bars represent standard deviations from two replicates.

The *K*_*M*_ and *V*_max_ values were determined from the Lineweaver–Burk plot shown in Figure 4. The *K*_*M*_ and *V*_max_ values of soluble and immobilized enzymes are given in Table 2, whereby the Michaelis constant (*K*_*M*_) shows the enzymatic affinity for its substrate, while the maximum velocity (*V*_max_) measures the extent of the activity [17]. Based on the data in Table 2, the decreasing *V*_max_ and increasing *K*_*M*_ trends of the immobilized enzyme were observed. The results showed that a higher *K*_M_ of the immobilized enzyme leads to a lower enzyme affinity which is caused by structural changes and lower accessibility of the substrate to the active site of the enzyme, thereby reducing *V*_max_ of the reaction [17, 24]. The immobilized enzyme showed a significant (*p* < 0.05) decrease in its reaction rate compared to the soluble enzyme. Also, it showed that immobilized enzymes needed higher substrate concentration to achieve *V*_max_. Furthermore, the bentonite structure exhibits an interlayer that entraps enzyme molecules and prevents the entrance of substrate. The hydroxyl groups (−OH) in the octahedral layer also strengthen noncovalent bonds between enzyme and bentonite [8, 25].

### 3.5. Reusability Study

**Figure 5:**
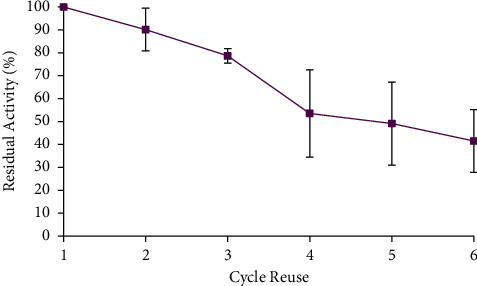
Reuse cycle of the immobilized enzyme. Assume the initial enzyme activity was 100%. The data are presented as mean ± SD; *n* = 2; a significant difference = *p* < 0.05. The error bars represent standard deviations from two replicates measurements.

One of the advantages of immobilized enzymes is that they are repeatedly used in new substrates. Furthermore, in the immobilization of enzyme molecules on the bentonite matrix, physical adsorption occurs; hence, the enzyme molecules do not have direct interaction with the substrate [23, 26, 27]. The enzymatic molecules that were entrapped on the matrix react with the new substrate until exhausted. Therefore, the reuse of immobilized enzyme led to the significant (*p* < 0.05) decrease of its activity due to repeated washing. The reuse cycle of immobilized enzyme is shown in Figure 5, and the result showed that the residual activity after six cycles of reuse was 42%.

### 3.6. Determination of Thermal Stability

**Figure 6:**
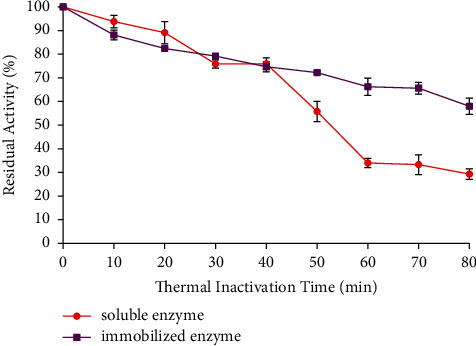
Thermal stability of the soluble and immobilized enzymes. The data were determined at each optimum temperature and each optimum starch concentration. Assume the initial enzyme activity was 100%. The data are presented as mean ± SD; *n* = 2; a significant difference = *p* < 0.05. The error bars represent standard deviations from two replicates measurements.

The thermal stability of soluble and immobilized enzymes is shown in Figure 6 where the soluble enzyme retained 29% of the residual activity after inactivation at 60 C for 80 min. Nevertheless, the residual activity of the immobilized enzyme was higher than the soluble, which has a retainment of up to 56%. The immobilized enzyme molecules were protected by the matrix from the effect of extreme temperature. As a result of this, the immobilized enzyme has higher thermal stability than the soluble. After immobilization, the immobilized enzyme showed a significant (*p* < 0.05) increase in thermal stability compared to the soluble enzyme.

### 3.7. Determination of *t*_1/2_, *k*_*i*_, and Δ*G*_*i*_

**Figure 7:**
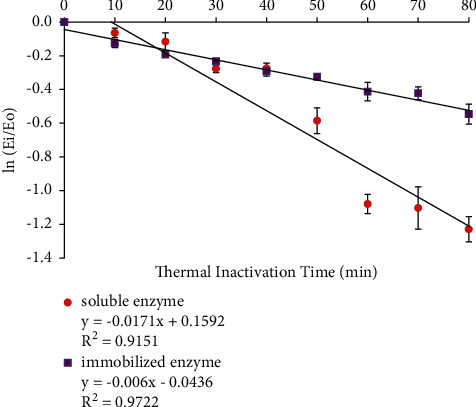
First-order inactivation rate plot of soluble and immobilized enzymes. The data are presented as mean ± SD; *n* = 2; a significant difference = *p* < 0.05. The error bars represent standard deviations from two replicates measurements.

The residual activities of soluble and immobilized enzymes from the determination of thermal stability were plotted to the first-order enzyme inactivation rate graph, as shown in Figure 7. The slope of graph ln(*E*_*i*_/*E*_*o*_) against *t*_*i*_ is expressed as the value of *k*_*i*_. The results in Figure 7 show that the *k*_*i*_ values of soluble and immobilized enzymes were 0.0171 and 0.0060 min^−1^, respectively. The decrease in the *k*_i_ value of immobilized enzyme leads to the decrease of denaturation rate which is due to the flexibility of the enzyme in water. Hence, the folding conformation in the immobilized enzyme structure increase for higher stability [21]. The thermal inactivation rate constant (*k*_i_) was used to calculate *t*_1/2_ and Δ*G*_*i*_ values, which are given in Table 3.

From Table 3, there is an observed significant (*p* < 0.05) increase in the half-life (*t*_1/2_) and Δ*G*_*i*_ of immobilized enzyme compared to the soluble enzyme. The half-life (*t*_1/2_) is the time required for the enzyme to break down the substrate until the enzyme loses half of its activity [23]. These results showed that the immobilized enzyme takes a longer time to be converted into the enzyme-substrate complex after which it loses its activity. In addition, the immobilized enzyme has higher Δ*G*_*i*_ which showed the increase of folding conformation in the enzyme structure. The increase in Δ*G*_*i*_ caused a more rigid and less flexible enzyme conformation in water. Hence, the energy required for denaturation becomes higher [21]. Based on the increase of half-life, the stability of the immobilized enzyme also increased approximately three-fold compared to the soluble. This study showed better results than the previous [10].

## 4. Conclusions

The stability of *α*-amylase enzyme from *A. fumigatus* had been improved by the immobilization method on a bentonite matrix. The increase in *t*_1/2_ and ΔG_i_ showed that the immobilized enzyme was more stable than the soluble. Furthermore, the improvement in the stability of *α*-amylase enzyme by immobilization on a bentonite matrix, based on increasing of its half-life, was three-fold higher than the soluble enzyme, while the residual activity of the immobilized enzyme after six cycles of reuse was 42%.

## Figures and Tables

**Table 1:** Summary of partial purification procedures of *α*-amylase from *A. fumigatus* unit activity was determined with 0.1% soluble starch as a substrate in aquadest (pH ± 6.5) at 60°C for 10 min.

Step	V (mL)	Unit activity (U/mL)	Total activity (U)	Protein content (mg/mL)	Total protein (mg)	Specific activity (U/mg)	Yield (%)	Purity
Crude enzyme	3500	100.37	351308.7	0.2123	742.91	472.88	100	1
Fraction (NH_4_)_2_SO_4_ (20–85%)	140	345.57	48379.7	0.0870	12.19	3970.08	13.77	8
Dialysis	239	179.68	42993.4	0.0398	9.52	4514.67	12.24	10

**Table 2:** *K*_*M*_ and *V*_max_ values for soluble and immobilized enzymes. The kinetic parameters were determined at each optimum temperature and the starch concentrations from 2.0 to 10.0 mg/mL. The values were shown as mean ± SD, *n* = 2.

Sample	*V*_max_ (*μ*mole mL^−1^ min^−1^)	*K*_*M*_ (mg mL^−1^ substrate)
Soluble enzyme	10.90 ± 1.894	3.04 ± 1.043
Immobilized enzyme	1.44 ± 0.299	8.31 ± 2.666

**Table 3:** *k*_*i*_, Δ*G*_*i*_, and *t*_1/2_ values for soluble and immobilized enzymes. The values are shown as mean ± SD, *n* = 2.

Sample	*k*_*i*_ (min^−1^)	*t*_1/2_ (min)	Δ*G*_*i*_ (kJ mole^−1^)	Stability improvement
Soluble enzyme	0.0171 ± 0.0008	40.53 ± 1.9001	104.47 ± 0.1296	1
Immobilized enzyme	0.0060 ± 0.0003	115.50 ± 5.7895	107.37 ± 0.1386	2.9

## Data Availability

The data used to support the findings of this study are available from the corresponding author upon request.
